# Study on Lyophilised Orodispersible Tablets from Plant-Based Drinks as Bulking Agents

**DOI:** 10.3390/pharmaceutics17020195

**Published:** 2025-02-04

**Authors:** Adrienn Katalin Demeter, Dóra Farkas, Márton Király, Zoltán Kovács, Krisztina Ludányi, István Antal, Nikolett Kállai-Szabó

**Affiliations:** 1 Department of Pharmaceutics, Faculty of Pharmacy, Semmelweis University, Hőgyes E. Street 7-9, 1092 Budapest, Hungary; demeter.adrienn.katalin@semmelweis.hu (A.K.D.); farkas.dora@semmelweis.hu (D.F.); kiraly.marton@semmelweis.hu (M.K.); ludanyi.krisztina@semmelweis.hu (K.L.); antal.istvan@semmelweis.hu (I.A.); 2 Department of Food Measurements and Process Control, Institute of Food Science and Technology, Hungarian University of Agriculture and Life Sciences, Somlói Street 14-16, 1118 Budapest, Hungary; kovacs.zoltan.food@uni-mate.hu; 3 Center for Pharmacology and Drug Research & Development, Semmelweis University, 1085 Budapest, Hungary

**Keywords:** orally disintegrating tablet, plant-based drinks, dysphagia, excipients, freeze-drying method, electronic tongue

## Abstract

**Background/Objectives:** Oral administration of active pharmaceutical ingredients (APIs) is the most commonly used route of administration. As dysphagia is a prevalent problem, the size of the swallowed dosage form could negatively influence patient adherence. Orally disintegrating tablets (ODTs) are beneficial dosage forms because they disintegrate within a few seconds in the oral cavity without water. Lactose is one of the most commonly used excipients in the pharmaceutical industry; it served as the central concept of a recent publication on the formulation of milk-based ODTs despite lactose malabsorption being widespread worldwide. Consequently, the plant-based alternative market has grown exponentially and has become a prevailing food trend, with various alternatives to choose from. For this reason, the development of a nonsteroidal anti-inflammatory drug (NSAID)-containing ODT with plant-based drinks (PBDs) was assessed for its innovative potential. **Methods:** Different PBDs were investigated and compared to traditional and lactose-free milk. The liquids’ viscosity, pH, and particle size were determined, and an electronic tongue was used for the sensory evaluation. The various ODTs were prepared with the freeze-drying method, and then the qualitative characteristics of the dosage form were investigated. **Results:** Our different measurements show that different plant beverages differ from each other and that these differences have an impact on the technological processing. According to the HPLC-DAD measurements, all values were in the required range. **Conclusions:** These measurements suggest that the soya drink is the most similar to traditional cow milk and would be the most appropriate choice among the investigated plant-based drinks to be used as a carrier system for an ibuprofen-containing ODT.

## 1. Introduction

Although oral administration is still one of the most frequently used routes of administration, most medicines are white, bitter tablets or capsules, and they can be hard to swallow due to their size or shape [1]. Dysphagia, or difficulty swallowing, is not only common in hospitals and long-term care facilities, but it is also a problem when the patient lives at home. Dysphagia occurs in all age groups, although it is more common among the elderly [2,3,4].

In the treatment of dysphagia patients, a variety of pharmaceutical technology options are now available to facilitate swallowing. One approach is to use conventional dosage forms but with special designs (e.g., filmcoating of the surface) [5]. Another option is the use of multiparticulate systems, such as sprinkle dosage forms, or an innovative solution in paediatrics could be to use medicated straws [6,7], but orally dispersible drug delivery systems are also a widely used solution. These include orodispersible granules, mini-tablets, and oral lyophilisates, along with drug strips [8,9]. Among these, orally dispersible tablets (ODTs) are the most commercially popular option [10]. ODTs could increase compliance in paediatric use (mainly for children and adolescents because they dissolve within minutes in the oral cavity upon contact with saliva) [11,12]. For treating pain and fever, paracetamol and the nonsteroidal anti-inflammatory drug (NSAID) ibuprofen (IBU) are the most used for this purpose. The recommended dosage of ibuprofen, administered orally for acute pain, is 10 mg/kg every 6 to 8 h, and the cumulative daily dose should not exceed 30 mg/kg. While other NSAIDs are also approved for use in children, ibuprofen is the only one suitable for infants as young as 3 months due to its proven efficacy and safety profile, although this depends on the dosage form and excipients [13].

There are many different methods for the production of ODTs, the most common industrial solutions today being compression using special fillers and superdisintegrants, as well as freeze-drying processes from aqueous systems. In addition to these processes, it is worth noting that the first FDA-approved ODT drug product made using 3D printing is Spritam, which contains levetiracetam [14]. As this new manufacturing process has appeared in ODT production, the number of new different excipients and bulking agents is expected to increase. Hygroscopic characteristics, along with the thermal and humidity sensitivity of ODTs, can influence their physical integrity, which can be modified with excipients and bulking agents [15]. However, in the case of ODTs, the use of excipients can improve patient compliance and acceptability due to masking flavours, improving processing, and optimising product performance [16]. In most medicines, excipients play a supportive role in delivering the active ingredient (API). Still, in some cases, excipients have more critical and complex roles; they can even be the main active ingredient [17,18].

Excipients are manufactured from various sources, including plants, animals, and minerals, as well as biotechnological and chemical synthesis [19]. The increased number of excipients required more regulation, so the International Pharmaceutical Excipients Council (IPEC) was formed. It has sought to standardise the purity and testing for functionality criteria. Global pharmaceutical market sales are rising, and the market value is also growing at a notable compound annual rate [20,21]. Milk, as an inexpensive carrier suitable for delivering active pharmaceutical ingredients, has been studied in numerous publications. A literature review by Salim and colleagues revealed that the use of milk and infant formulas can favourably influence the solubility and bioavailability of active ingredients and is suitable for the preparation of various pharmaceutical dosage forms [22]. Iurian and colleagues prepared loratadine-containing lyophilisates using freeze-drying from milk with different fat contents and an infant formula [18]. Even though lactose malabsorption is widespread in most of the world, with wide variation between regions and, to some extent, also within countries [23,24], lactose is one of the most commonly used excipients in the pharmaceutical industry [25,26]. The overall estimated frequency of lactose malabsorption is around two-thirds of the world’s population [27].

Considering the aforementioned reasons, the plant-based alternative market is growing exponentially and has become a prevailing food trend, which has led to the creation of many novel beverages from cereals, legumes, nuts, seeds, and pseudocereals [28]. Each variety has unique characteristics in terms of flavour, texture, and nutritional composition, offering consumers a diverse range of choices tailored to meet individual preferences and dietary needs. The PBDs that are sweet, creamy, smooth, nutty, and white are preferred by the consumers, while aftertaste, brown colour, beany sensation, watery consistency, and off-flavour reduce favourability [29,30]. They can be consumed in pure form or used as a companion for coffee and tea and can serve as an ingredient in processed foods or, as in this case, as a bulking agent for an active ingredient [31].

The aim of this study was to compare the pharmaceutical properties of plant-based beverages as a raw material for orodispersible tablets with those of previously reported orodispersible carrier systems prepared using milk. To achieve this, five types of commercially available plant-based drinks and one formula were analysed, and solid dosage forms suitable as carrier systems were prepared using freeze-drying. We evaluated the pharmaceutically relevant properties, with a particular focus on critical quality attributes for orodispersible systems, such as disintegration time and taste perception.

## 2. Materials and Methods

### 2.1. Materials

The active ingredient ibuprofen was purchased from Merck (Merck KGaA, Darmstadt, Germany). The used drinks were purchased locally: the plant-based drinks were from the Alpro brand (Alpro Ltd., Ghent, Belgium), the dairy products were Mizo (Sole-Mizo Zrt., Szeged, Hungary), and the formula was a particular sugar-free type, Nutricia Nutridrink Diacare (Danone Kft., Budapest, Hungary). The kinds of milk, formula, and plant-based drinks were coded as follows: LFM (lactose-free milk 1.5%), M3.5 (Mizo milk 3.5%), M1.5 (Mizo milk 1.5%), F (formula: Nutricia Nutridrink Diacare Vanilla flavour), S (soya), H (hazelnut), R (rice), C (coconut), and A (almond).

### 2.2. Preliminary Studies of the Milk, Plant-Based Drinks, and Formula

The first part of this study was conducted with nine types of drinks (milk, plant-based drinks, and formula). The preliminary study aimed to compare the nutritional values of the beverages.

#### 2.2.1. Viscosity

The viscosity of the nine drinks (before freeze-drying) was measured using a Fluidicam^TM^ RHEO viscosimeter (Microtrac Formulaction, Toulouse, France), which is based on microfluidic principles with optical acquisition, which could be used to determine the viscosity of different kinds of liquids or even gels [32]. An appropriate protocol was chosen: the temperature was set to 25 °C, a 150 μm plastic chip was used, the shear rate was set to 1000–5000 s^−1^, 5 points per curve were taken, and each point was calculated from 10 measurements. The proper reference solutions were chosen. These liquid references, purchased from the producer, had different viscosities, with targets of 5, 50, and 500 mPas at 25 °C. For the beverages, the appropriate viscosity was proven to be 50 mPas.

#### 2.2.2. pH

The pH meter (serven Compact S220, Mettler-Toledo Kft., Budapest, Hungary) was calibrated against buffer solutions of known hydrogen ion activity. The glass probe was put into 50 mL solution. The pH was measured at room temperature, with three parallels.

#### 2.2.3. Particle Size Determination

Particle size analysis was performed using the laser diffraction method. The particle size of the fresh, unaltered liquid samples was measured at 25 °C using a MasterSizer 2000^TM^ (Malvern Instruments Ltd., Malvern, UK) connected to a Hydro SM manual liquid sample dispersion unit. Laser diffraction analyses the angular distribution of light scattered by a diluted sample (0.1 mL dispersed in 100 mL demineralised water), allowing for the detection of particles ranging in size from 0.1 to 3000.0 µm. Each sample was measured three times individually. According to recommendations from the Malvern diffraction application, each measurement lasted 20 s to ensure slow-moving larger aggregates could pass through the detector array.

#### 2.2.4. Sensory Valuation with Electronic Tongue

An electronic tongue (Alpha Astree, Alpha M.O.S., Toulouse, France) was used for the sensory evaluation measurements. The Alpha Astree electronic tongue models how the human tongue works. It is designed to analyse, recognise, and identify complex dissolved organic and inorganic components. The equipment must also learn the different reference flavours, just like a human does. However, once trained, it can be used to identify unknown products based on their flavour. The measurement result, known as a “fingerprint”, provides the opportunity to compare the tested samples by their general taste profile. The device contains seven special sensors, in this case, sensors developed for food testing. This measurement method allows qualitative and quantitative determination and provides an objective comparison [33]. In the electronic tongue studies, milk and PBD samples were analysed at a concentration of 10 mL/100 mL (10-fold) dilution in distilled water. At the beginning of the session, the electronic tongue was conditioned by alternating between 0.01 M HCl and distilled water according to the instrument manufacturer’s instructions. A second calibration was performed with mixtures of the same proportions as the tested samples.

The first part of the experiment was conducted with the nine drinks to compare them and determine which is the most similar to traditional dairy (cow) milk. The second part of the experiment was carried out with lactose-free milk, soy, hazelnut, and coconut plant-based beverages, and these four with the API to compare the flavour, with or without the API.

### 2.3. Formulation of the ODTs

#### 2.3.1. Preparation of ODT

The drinks were stored at 4 °C before the experiment. The packaging was unsealed at the same time. To formulate the placebo ODTs, 1.5 mL liquid was poured into aluminium blisters for freeze-drying. The round blisters’ volume was approximately 1.7 mL, the diameter was 2.2 cm, and the depth was 0.5 cm. For the stock solution, the API (ibuprofen sodium salt) was dissolved into the selected type of drinks, and then it was poured into the blisters. For the ODTs contained 100 mg API, the dosage was chosen based on the solubility of the ibuprofen sodium salt, which is 100 mg/mL [34]. Moreover, the produced ODTs are designed for paediatric use in the first case, so a lower dosage is required.

#### 2.3.2. Freeze-Drying

**Figure 1:**
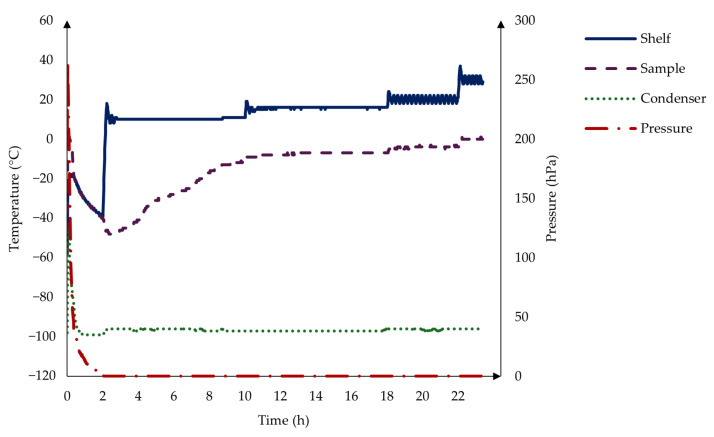
The freeze-drying process consists of a 2 h freezing stage at −40 °C, followed by 8 h drying at 10 °C, 8 h at 15 °C, 4 h at 20 °C, and 2 h at 30 °C in a vacuum.

The prepared samples in the blisters were subjected to the freeze-drying process [35,36] (Scanvac Coolsafe^TM^, LaboGene, Denmark), 66 samples at a time. The procedure consisted of a 2 h freezing stage at −40 °C, followed by 8 h drying at 10 °C, 8 h at 15 °C, 4 h at 20 °C, and 2 h at 30 °C in a vacuum, as you can see in the graph below in Figure 1. After the freeze-drying, the samples were stored in plastic bags with a desiccator to prevent moisture uptake.

### 2.4. Characterisation of the ODTs

The pharmaceutical characterisation measurements were carried out with placebo ODTs made from the nine drinks after freeze-drying.

#### 2.4.1. Uniformity of Mass

Based on the European Pharmacopoeia monograph [37], the masses of the nine types of drink-based placebo ODTs were measured on an analytical balance (Kern ABJ-NM/ABS-N, Kern & Sohn GmbH, Balingen, Germany); 20 orodispersible tablets were used for each batch.

#### 2.4.2. Disintegration Time

The disintegration time was measured according to the method described in the European Pharmacopoeia [38]. Six samples from each type were placed in cylindrical baskets, with a disc on top of them, and then the basket apparatus (Erweka^TM^ ZT4 Timer, ERWEKA GmbH, Langen, Germany) was started, and the cylindrical vessels were sunk into a beaker filled with 800 mL of water. The beaker was heated to 37 ± 0.5 °C with a water bath. The ODTs were placed in a dry basket at the beginning of each test. The time was recorded using a digital stopwatch until the tablets disintegrated completely.

#### 2.4.3. Residual Water Content of Freeze-Dried ODTs

The water content of the freeze-dried samples was determined using a Karl Fischer titrator (787 KF Titrino, Metrohm AG, Herisau, Switzerland). The measurement principles were as described in the European Pharmacopoeia [39]. Prior to the measurements of the samples, the water equivalency factor of the titrant (Aquastar^®^ CombiTitrant 5; Merck KGa, Darmstadt Germany) was determined using ultrapure MilliQ water (18.2 MΩ·cm at 25 °C; Simplicity^®^ UV Water Purification System, EMD Millipore Corporation, Billerica, MA, USA) (MQ water). The solvent was methanol (Aquastar^®^ CombiMethanol, max. 0.01% H_2_O; Merck KGa, Darmstadt Germany), which was titrated with Karl Fischer reagent before the measurement. An amount of 0.05 mg of ODT samples was accurately weighed, dispersed (1 min at 15,000 rpm), and then titrated with the reagent. Five parallel measurements were carried out for the evaluation.

#### 2.4.4. Study of the Effect of the Environment on the Structure

First, the ODTs’ images were taken immediately (50 MP, f/1.8, OIS (Optical Image Stabilisation), Samsung, Suwon, South Korea). Then, the tablets were left uncovered for 24 h, and a picture was taken again. The average temperature was 26 ± 2 °C, and the relative humidity was 60 ± 5%.

#### 2.4.5. Uptake of Methylene Blue Water

A simulated wetting test can be used to determine the wetting time of ODTs. Although numerous variations of the test are currently in practice, a standardised approach has yet to be established [40,41,42]. A typical wetting test includes putting an ODT on a coloured, wet filter paper and then recording the colour diffusion on the tablet. Based on previous studies, this method was developed to be the most appropriate one to test and analyse the tablets.

A Petri dish was filled with 16 mL 1 *w*/*w*% methylene blue solution and covered with filter paper, with a diameter of 7 cm. The Petri dish height was 1 cm, and the diameter was 6 cm. The ODT was placed on top of the filter paper, and the change was recorded for 3 min with a digital camera (50 MP, f/1.8, OIS (Optical Image Stabilisation), Samsung, Suwon, South Korea). At given times, image analysis was carried out with ImageJ (Wayne Rasband, National Institute of Health, Bethesda, MD, USA) [43] to measure the percentage and the speed of the uptake. The sampling times were the following: 0, 15, 30, 45, 60, 75, 90, 105, 120, 135, 150, 165, and 180 s.

#### 2.4.6. Determination of API Content with HPLC-DAD

Sample preparation was achieved by simple protein precipitation. For the preparation of calibration samples and QC samples in the surrogate matrix, 100 µL of spiking standard solution and 10 µL of internal standard working solution were mixed with 890 μL of the surrogate matrix. The surrogate matrix was prepared by dissolving one placebo ODT in 10 mL MilliQ water, and it was homogenised with a magnetic stirrer for 2 min at room temperature. In the study samples, 100 µL of water and 10 µL of internal standard (IS) working solution were mixed with 890 µL of the dissolved sample. For the dissolved samples, 1 ODT was dissolved in 10 mL water. For protein precipitation, 100 µL of trifluoroacetic acid was added to each sample and vortex-mixed. After centrifugation at 14,000 g for 10 min, aliquots (150 µL) of the supernatant were transferred to autosampler vials. Each time, ten samples were measured, with three repetitions.

Chromatographic separations were performed on an Agilent Series 1100 LC system (Agilent Technologies, Santa Clara, CA, USA). The analytes were separated on a C18 Zorbax Eclipse 100 Å column (4.6 mm × 150 mm, 5 μm). The column and the autosampler were maintained at 25 °C. An amount of 10 μL of sample was eluted under isocratic conditions over 5 min at a 2 mL/min flow rate. The mobile phase was composed of pH = 3 phosphate buffer and chromatography-grade methanol (30:70, *v*/*v*%). The detection was carried out with a UV-DAD detector at 225 nm.

The quantity of the substance was calculated by applying a predetermined calibration curve. The calibration standards were diluted from the stock solution to obtain five calibration levels and were run in duplicate at the beginning of each measurement process. The lowest and highest points of the calibration curve coincided with the lower limit of quantitation (LLOQ, 0.625 mg/mL) as well as the upper limit of quantitation (ULOQ, 10 mg/mL). Intra-day accuracy and precision were assessed by evaluating five replicates of QC (10 mg/mL) samples (*n* = 5). Accuracy was expressed as a percentage of the nominal concentration, and precision was calculated as the relative standard deviation (RSD). The acceptance criteria for both parameters were set at ±5% [44].

The nominal active substance content of the sample was 8.9 mg, but from the measurement, an average content was calculated. According to the European Pharmacopoeia, “2.9.6. Uniformity of content of single-dose preparations”, the requirements are that each individual content is between 85 and 115% of the average content; only one can be outside this range but has to be between 75 and 125% and none are allowed to be outside the limits of 75-125% of the average content [45].

## 3. Results and Discussion

There are several types of milk and plant-based beverages. However, they differ in physical properties, so the applicability of freeze-drying as a carrier system for the active ingredients is also different. Traditional dairy (cow) milk has long been used and explored as an excipient, but more and more people cannot fully digest lactose. Hence, the need for a lactose-free alternative is increasingly justifiable. Plant-based beverages are receiving more attention. There are several flavours to choose from, but it is a question of which one could be used as a bulking agent. The most significant advantage of an ODT is that it can be swallowed easily, which is helpful, especially in the paediatric field. For this, the structure needs to be porous, but it also needs to have the required mechanical strength. This study focused on the selection of the most useable PBD for ODTs and for the accurate active ingredient (API) dose, so it is also important how good the taste coverage is. The chosen API was ibuprofen, which is a widely used non-steroidal anti-inflammatory drug that can also be used for children; on the other hand, it has a bitter taste. That is the reason why we selected these samples.

### 3.1. Results of the Preliminary Studies of Milk, Plant-Based Drinks, and Formula

The first part of this study was conducted with nine types of drinks containing milk, plant-based drinks, and a formula, with the composition described in Table 1. The collected data are from the producer.

For protein, the soya drink has the highest protein content, similarly to cow milk. Soy-based plant drinks are widely regarded [31,46,47,48] as a complete protein source for adults, providing all essential amino acids. The others have lower content, with rice and coconut having the lowest.

The PBDs contain dietary fibres, which refer to non-digestible carbohydrates. Dietary fibres retain their structural integrity as they move through the digestive tract because the digestive enzymes in the human body do not break them down. In PBDs, various soluble or insoluble dietary fibres present in the cell walls of seeds or cereals, such as almond polysaccharides and soy polysaccharides, exhibit potential prebiotic characteristics. Nonetheless, this prebiotic function is absent in dairy milk. Soy drinks enriched with fibre can reduce plasma cholesterol levels in both animals and humans without interfering with the absorption of essential minerals such as zinc and copper. Additionally, they support gut health and help regulate blood sugar and lipid levels [31,49].

Plant-based drinks are rich in unsaturated fatty acids, with typically low amounts of saturated fats and no cholesterol. This nutrient profile helps reduce low-density lipoprotein (LDL) and overall cholesterol levels, offering significant benefits for individuals managing high blood cholesterol and cardiovascular diseases.

Lactose is the main sugar contained in milk and dairy products, which can lead to lactose malabsorption. On the other hand, PBDs are lactose-free, but the original, sugar-containing ones were compared because the taste masking property is needed for this research.

Overall, the data presented above show that the composition of PBDs is significantly different from dairy milk. Due to this difference in lipid ratio and composition compared to milk, the solubilisation efficiency of the active substances may be different, which may have an impact on the in vivo behaviour. However, it is important to note that certain PBDs may cause allergic reactions (soy and almonds) [50].

### 3.2. Viscosity Results

**Figure 2:**
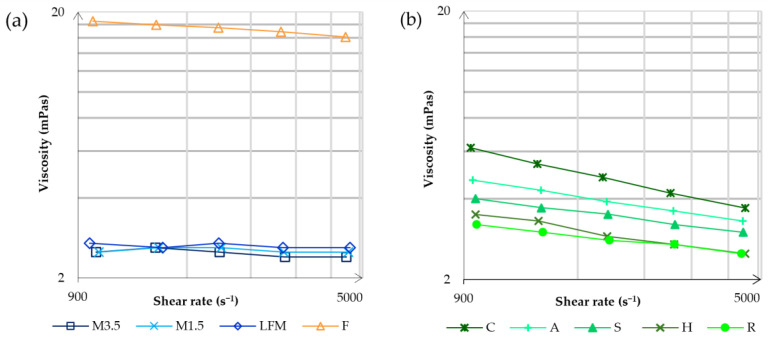
The viscosity measurements by the Fluidicam™ RHEO microfluidic viscometer. The results of the dairy milk and formula are shown in (**a**), and then the results of the PBDs are shown in (**b**). LFM (lactose-free milk 1.5%), M3.5 (Mizo milk 3.5%), M1.5 (Mizo milk 1.5%), F (formula: Nutricia Nutridrink Diacare Vanilla flavour), S (soya), H (hazelnut), R (rice), C (coconut), and A (almond).

The viscosity was measured by a Fluidicam™ RHEO microfluidic viscometer (Microtrac Formulaction, Toulouse, France). The adjustable shear range depends on the selected microchip and the rheological properties of the sample. The results of the dairy milk and formula are shown in Figure 2a, and the results of the PBDs are shown in Figure 2b.

It is apparent that the formula has the highest viscosity, significantly higher than dairy milk. A reason could be that the formula is primarily designed to supplement or replace nutrition, so it needs to contain many nutrients. Dairy milks have the lowest viscosity, and it is mostly the same at different shear rates. On the other hand, PBD viscosity decreases at lower shear rates. According to the viscosity, the most alike to dairy milk are the rice and hazelnut drinks; the most remote is coconut.

### 3.3. pH Results

**Figure 3:**
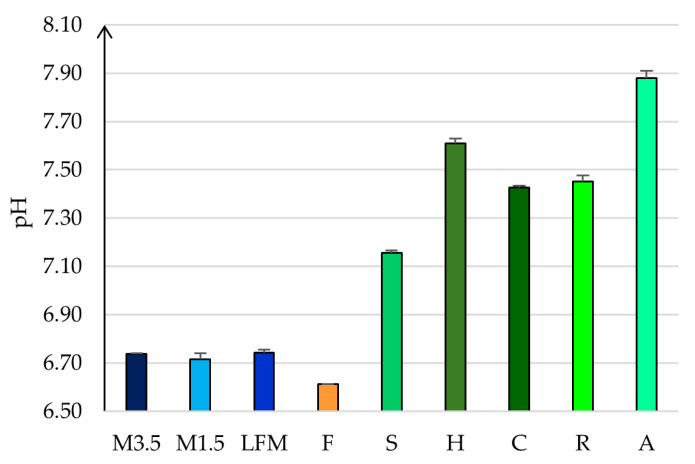
The measured pH values. LFM (lactose-free milk 1.5%), M3.5 (Mizo milk 3.5%), M1.5 (Mizo milk 1.5%), F (formula: Nutricia Nutridrink Diacare Vanilla flavour), S (soya), H (hazelnut), R (rice), C (coconut), and A (almond).

The measured pH values are shown in Figure 3. Dairy milks have a lower pH of 6.7. However, the PBDs have a more basic pH value between 7 and 8. The soya drink has the lowest, 7.2 ± 0.01, so this is the most alike to the dairy milk, and the almond has the highest value, 7.88 ± 0.03. The pH could slightly differ, depending on the tested brands [51].

### 3.4. Particle Size

**Figure 4:**
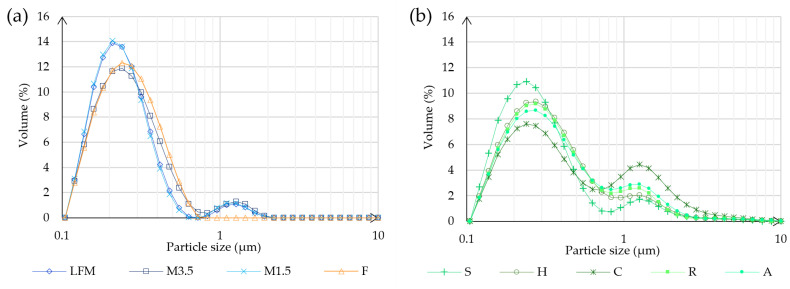
Particle size analysis result. (**a**) Shows the results of the different kinds of milk and the formula. (**b**) Shows the results of the PBD samples. LFM (lactose-free milk 1.5%), M3.5 (Mizo milk 3.5%), M1.5 (Mizo milk 1.5%), F (formula: Nutricia Nutridrink Diacare Vanilla flavour), S (soya), H (hazelnut), R (rice), C (coconut), and A (almond).

According to the dynamic light scatter (DLS) measurements in Figure 4, the hydrodynamic diameter was determined. The particle size is separated into two main groups in both cases, except for the formula, because all particles fall within the 0.1–0.7 μm range. In case of the dairy milk, most of the particles are in the range of 0.10–0.7 μm and some bigger particles are in the range of 0.8–2 μm. However, the majority of the PBD particles are also in the range of 0.1–0.7 μm, but it is a lower volume because there are more in the range of 0.8–5 μm. This could be because of the different manufacturing process, which can lead to some remaining bigger particles. The most similar to cow milk is soya, which is prepared by fermentation.

### 3.5. Electronic Tongue

Electronic tongues have emerged as a valuable solution, with their versatile designs, rapid operation, and real-time data processing capabilities. An electronic tongue has proven its usefulness in food products, water samples, and taste masking technologies for pharmaceuticals, and it can also be used with other detectors [52,53,54]. It can be used to fingerprint food properties and to control food production from the first steps until the shelf [33]. It is essential to analyse the results using the proper method [55,56]. It is a system that usually consists of an array of non-specific chemical sensors combined with appropriate data acquisition systems and chemometric tools [57].

During the evaluation, multivariate statistical methods are typically applied as pattern recognition algorithms. Their use is necessary due to the multiple simultaneously operating working electrodes.

**Figure 5:**
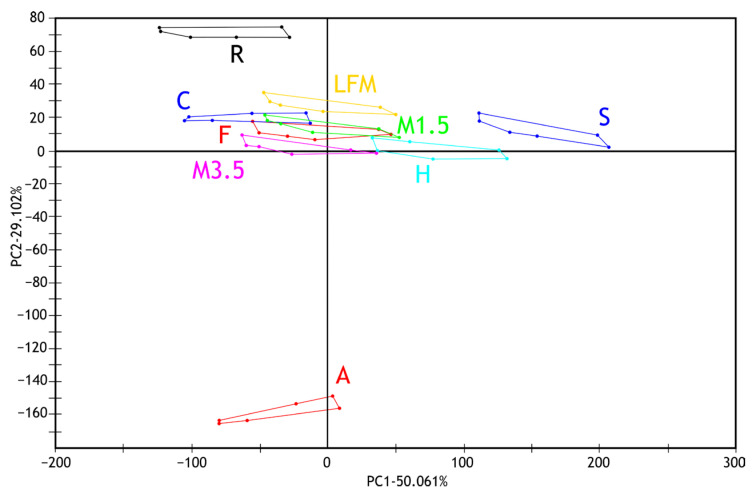
PCA results (PC1-PC2) of electronic tongue measurements of milk and PBD solution samples tested. LFM (lactose-free milk 1.5%), M3.5 (Mizo milk 3.5%), M1.5 (Mizo milk 1.5%), F (formula: Nutricia Nutridrink Diacare Vanilla flavour), S (soya), H (hazelnut), R (rice), C (coconut), and A (almond).

The principal component analysis (PCA) results of the electronic tongue measurement of the tested milk and PBD sample solutions are presented in Figure 5 for the first two principal components. Along with the first principal component (PC1—50.061%), the measurement points of the soy drink and hazelnut drink samples are the most different from the tested sample groups. Along the second principal component (PC2—29.102%), the almond and rice drink groups show separation from the others. The milk 1.5% sample group overlaps with the formula and coconut drink measurement points along the first two principal components. Also, it shows similarities with the milk 3.5% and lactose-free milk sample groups.

**Figure 6:**
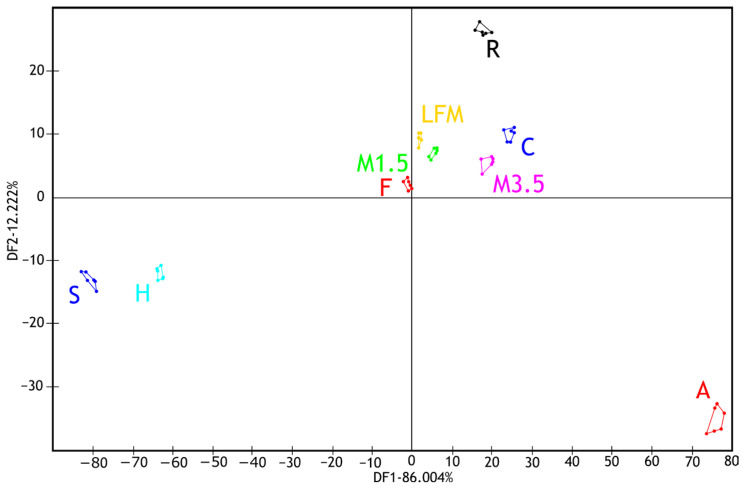
LDA results of the electronic tongue measurements for the tested dairy milk and PBD solutions. LFM (lactose-free milk 1.5%), M3.5 (Mizo milk 3.5%), M1.5 (Mizo milk 1.5%), F (formula: Nutricia Nutridrink Diacare Vanilla flavour), S (soya), H (hazelnut), R (rice), C (coconut), and A (almond).

Figure 6 shows the results of the discriminant analysis (LDA) of the electronic tongue measurements for the tested dairy milk and PBD samples based on the discrimination of sample types. Despite the overlapping groups observed in the PCA, all sample groups are well separated in the LDA plot. Like the observations from the PCA, it can be noted that the milk samples (1.5%, 3.5%, and lactose-free) and the formula group are located close to each other. These are followed by groups of coconut and rice drinks. The greatest distance is observed for the hazelnut and soy drink groups, followed, in the opposite direction, by the almond drink group.

**Figure 7:**
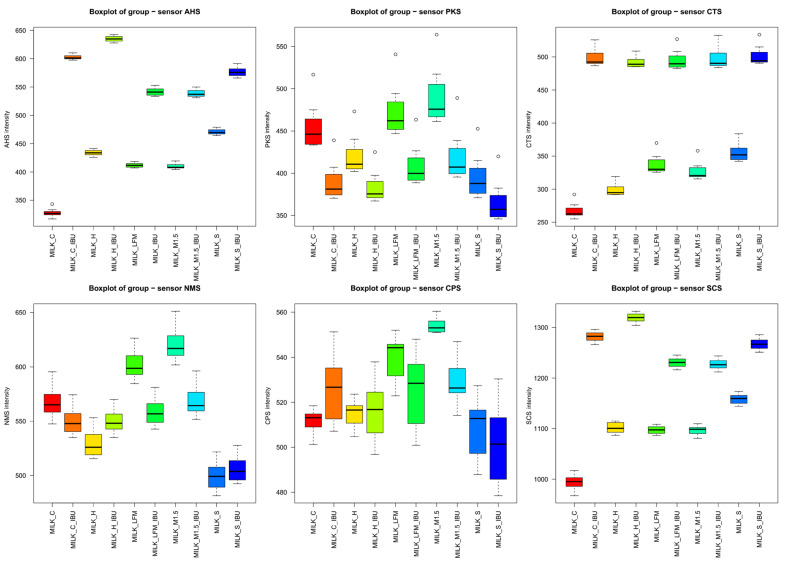
Boxplot results of six selected sensors of the electronic tongue for the tested milk and plant-based drink solutions. (_IBU means ibuprofen-containing samples). AHS (Average Histogram Signal), PKS (Peak Signal), CTS (Computed Taste Score), NMS (Normalized Measurement Signal), CPS (Cycles Per Second), SCS (Sensor Contribution Score).

After these evaluations, five samples were selected to see the taste masking effectiveness of the samples when they contained ibuprofen. Figure 7 illustrates the results of the electronic tongue sensors’ boxplots and ANOVA p-values for the tested milk and PBD solutions and with ibuprofen to compare, focusing on six selected sensors. Even at the individual sensor level, the samples are well distinguished. The ANOVA analysis shows significant differences across all seven sensors. According to the sensor AHS, CTS, and SCS, the two groups are separated nicely, the ones with ibuprofen and without. Also, it is recognisable that the samples are capable of taste masking.

**Figure 8:**
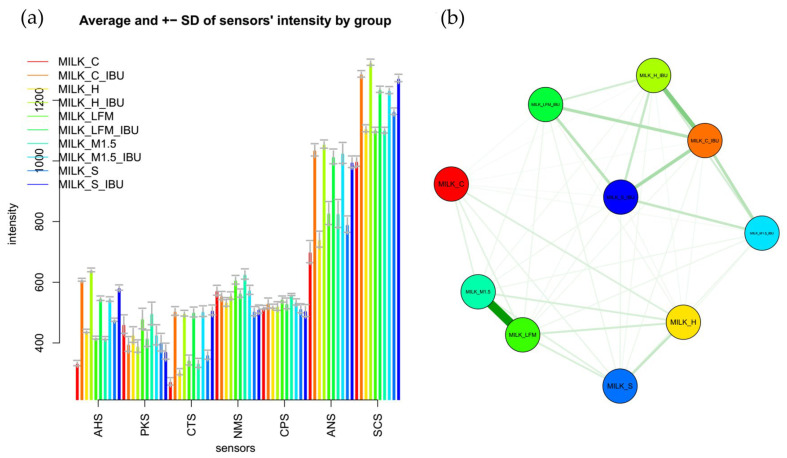
Mean sensor values of the electronic tongue for the tested dairy milk and plant-based drink solutions (**a**) shown per sample; (**b**) comparative chart based on the multidimensional group distances of the tested samples. LFM (lactose-free milk 1.5%), M3.M1.5 (Mizo milk 1.5%), S (soya), H (hazelnut), C (coconut), and and “_IBU” means ibuprofen-containing samples. AHS (Average Histogram Signal), PKS (Peak Signal), CTS (Computed Taste Score), NMS (Normalized Measurement Signal), CPS (Cycles Per Second), ANS (Average Normalized Signal), SCS (Sensor Contribution Score).

Figure 8a shows the mean values of the electronic tongue sensors, broken down by sample, along with their standard deviations. Similar to the observations from the boxplot analysis, the mean sensor signal results, illustrated with error bars, clearly demonstrate that significant differences were observed between most samples, even at the sensor level.

Figure 8b presents a comparative analysis based on the multidimensional group distances of the tested samples. According to their multidimensional distances, the sample groups are separated into two main clusters: one group included samples without ibuprofen, while the other group of samples containing ibuprofen. In Figure 8b, groups of samples connected by thicker lines and positioned closer together indicated more remarkable similarity based on the electronic tongue results. In contrast, the electronic tongue perceived groups located farther apart and connected by thinner lines as less similar.

**Figure 9:**
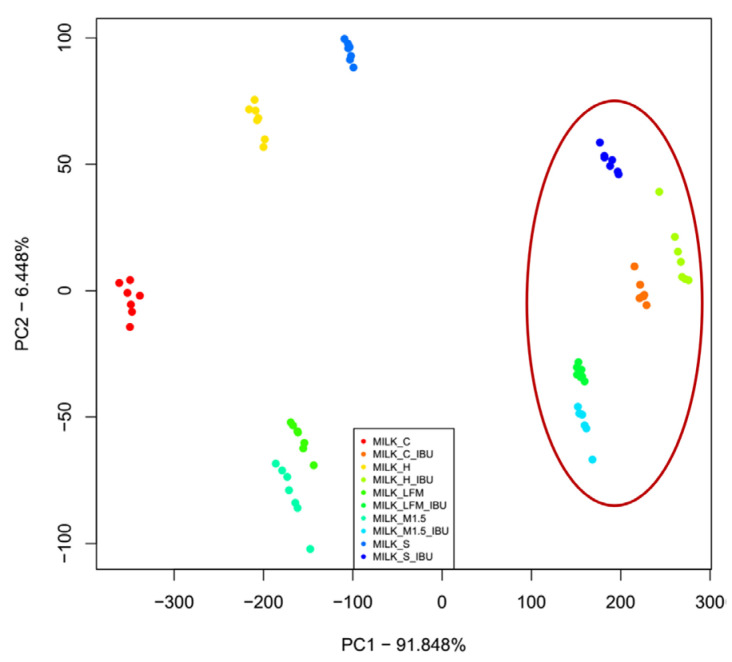
The principal component analysis (PCA) results of the electronic tongue measurements for the tested dairy milk and plant-based drink solutions are shown: PC1-PC2 score plot. LFM (lactose-free milk 1.5%), M1.5 (Mizo milk 1.5%), S (soya), H (hazelnut), C (coconut), an the red circle and “_IBU” means ibuprofen-containing samples.

Figure 9 presents the results of the PCA measurements for the tested samples, focusing on the first two principal components. Along the first principal component (PC1—91.85%), and the second (PC2—6.49%), those without ibuprofen and those containing ibuprofen are most distinctly separated. Moreover, all the tested sample groups were well separated.

**Figure 10:**
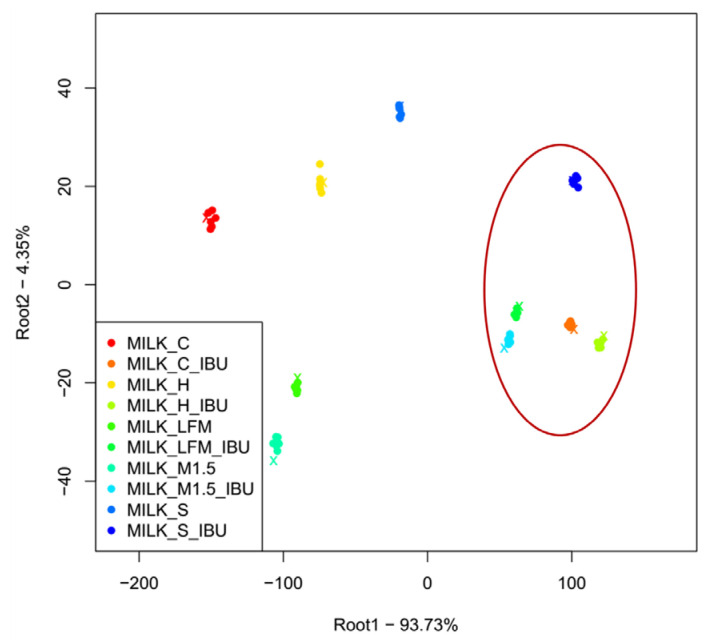
The LDA results of the electronic tongue measurements for the tested milk and plant-based drink solutions. (Lactose-free milk 1.5%), M1.5 (Mizo milk 1.5%), S (soya), H (hazelnut), C (coconut), and); the red circle and “_IBU” means ibuprofen-containing samples.

Figure 10 presents the results of the LDA measurements for the samples based on the discrimination of sample types. Like the PCA results, the samples containing ibuprofen are clearly separated from those without ibuprofen. The milk and lactose-free milk sample groups are well-separated from the other groups, the PBDs, and positioned closely to each other, whether ibuprofen is present or not.

The LDA classification models achieved 100% recognition and prediction accuracy, meaning that each sample was flawlessly classified based on the electronic tongue measurement results.

### 3.6. Pharmaceutical Characterisation of the ODTs

#### 3.6.1. Mass, Water Content, and Disintegration Time

Table 2 summarises the measured mass, water content, and disintegration time.

The formula has the highest mass, and this is the only one that disintegrates in more than five minutes. This could be the result of its high viscosity and increased nutrition content, resulting in high density. The PBDs have lower mass; associated with this, the disintegration time is also quicker than that of the dairy milk ODT. Nonetheless, the disintegration time of all the samples (except the formula mentioned before) is under five minutes, which falls into the requirements of the European Pharmacopoeia [38]. Regarding the water content, all samples had less than 5%.

#### 3.6.2. Effect of the Environmental Factors on the Structure

**Figure 11:**
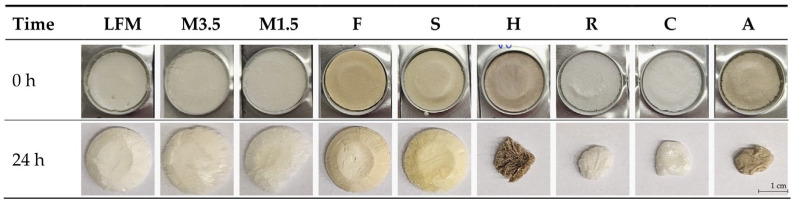
Structural change due to environmental factors (26 ± 2 °C, 60 ± 5% Rh). LFM (lactose-free milk 1.5%), M3.5 (Mizo milk 3.5%), M1.5 (Mizo milk 1.5%), F (Formula: Nutricia Nutridrink Diacare Vanilla flavour), S (soya), H (hazelnut), R (rice), C (coconut), and A (almond).

The effect of the storage conditions (26 ± 2 °C, 60 ± 5% Rh, 24 h) on the structure is variable; they affect the PBDs more than the cow milk ones. Also, it is spectacular to see how the structure changes, as shown in Figure 11.

The dairy milk, formula, and soya PBD remain the same, but the other PBD samples shrink. According to this, the soya is the most similar to the dairy milk samples; the other PBDs should be taken immediately before they absorb moisture and change shape because this can influence patient adherence.

#### 3.6.3. Results of the Methylene Blue Water Uptake

**Figure 12:**
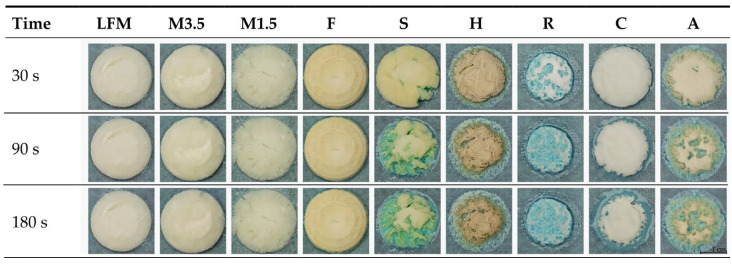
The uptake of methylene blue water. LFM (lactose-free milk 1.5%), M3.5 (Mizo milk 3.5%), M1.5 (Mizo milk 1.5%), F (formula: Nutricia Nutridrink Diacare Vanilla flavour), S (soya), H (hazelnut), R (rice), C (coconut), and A (almond).

The uptake of methylene blue water shows how fast and how much water the tablets can uptake if they are just placed on wet paper, like when the patients place it on their tongues. Figure 12 shows that the PBDs can uptake the blue water; the coconut one is the fastest, but the dairy milk and formula are not wetted easily in this model environment. This could be because of the thin water layer, the fact that dairy milk drinks contain many fatty acids, and the fact that the formula is too dense and heavy.

**Figure 13:**
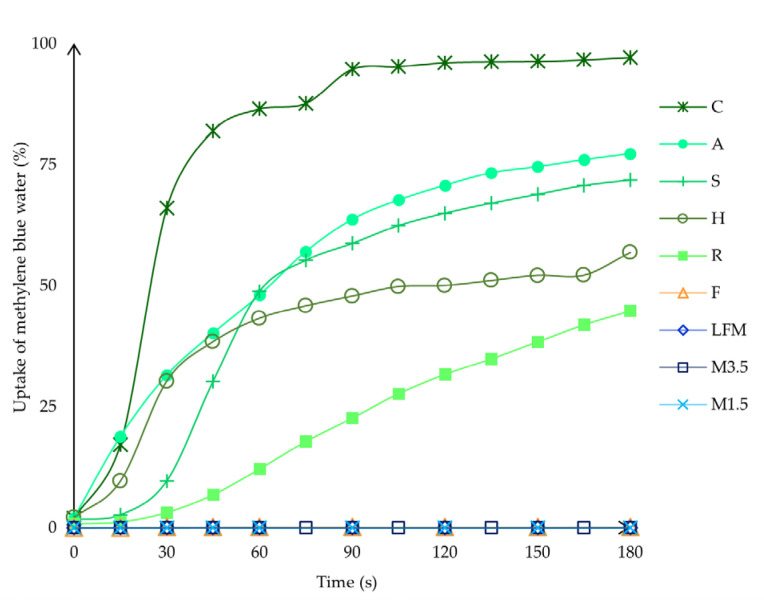
The water uptake capability in percentage was analysed with ImageJ. LFM (lactose-free milk 1.5%), M3.5 (Mizo milk 3.5%), M1.5 (Mizo milk 1.5%), F (formula: Nutricia Nutridrink Diacare Vanilla flavour), S (soya), H (hazelnut), R (rice), C (coconut), and A (almond).

The water uptake capability was also analysed with ImageJ to see the exact process in numbers. With ImageJ, the blue and the original colour area were measured, and then the blue area percentage was calculated compared to the original whole area. According to these data, as shown in Figure 13, the coconut ODT is the fastest, nearly taking up to 100% in 90 s; only some parts remain white. This could be because the coconut ODT is highly porous and light. One should be aware, as it could take up the moisture from the fingers just by touching it, which could be a problem. The almond and soya are similar, taking up the blue water to 75%. Then, finally, the hazelnut takes up the blue water to 56% and the rice to 45%. However, the soya can take up the water quickly and efficiently and disintegrate in under 3 min. On the other hand, even after 24 h of being left outside, the structure remains intact, which is beneficial.

#### 3.6.4. API Content Determined with HPLC-DAD

The HPLC measurement results are in Table 3. According to this, all the samples meet the pharmacopoeial criteria, and the average API content is between 85 and 115%. The validation parameters for the method can be seen in the table, as all the parameters are within the range.

## 4. Conclusions

The large number of patients with dysphagia and the extensive variety of orodispersible products available on the market underline the continued importance of studies on orodispersible tablet formulations. Advances in pharmaceutical technology leverage increasingly new excipients and innovative methods to design patient-centric drug delivery systems. Lyophilised cow’s milk has been successfully applied as a drug delivery matrix in previous studies.

The successful development of plant-based beverage ODT systems as a formulation method may also be essential for paediatric medicines, as excipients safe for children may not always match the inactive components used in adult medicines. For plant-based drinks that do not trigger allergic reactions in children, and for younger children (such as newborns and infants), lactose-free infant formulae produced under strict conditions can be consumed in larger quantities than food and should be considered as potential carrier systems. The specific lipid composition of plant-based beverages and formulae may influence the solubilisation and taste perception of the active ingredient, differing from the properties of lyophilised orodispersible tablets produced using traditional non-complex aqueous systems.

In this study, we explored whether plant-based beverages could serve as suitable alternatives for developing orally dispersible drug delivery systems, particularly addressing the needs of patients with lactose intolerance. Several commercially available plant-based drinks (soya, hazelnut, rice, coconut, and almond) were examined, and successful freeze-drying processes were performed. Lyophilisates with various compositions were analysed and compared to milk-based lyophilisates, which have been previously demonstrated as effective drug delivery systems. Our findings indicate that the soya drink is the most promising candidate among the plant-based beverages evaluated in this study. Electronic tongue measurements revealed that soya beverages possess excellent taste masking properties, effectively masking the bitterness of ibuprofen. Additionally, the performance of the soya-based orodispersible system was comparable to that of traditional milk-based systems.

## Figures and Tables

**Table 1:** The nutritional values of the nine types of drinks, according to the producer. LFM (lactose-free milk 1.5%), M3.5 (Mizo milk 3.5%), M1.5 (Mizo milk 1.5%), F (formula: Nutricia Nutridrink Diacare Vanilla flavour), S (soya), H (hazelnut), R (rice), C (coconut), and A (almond).

g/100 mL	Milk			Formula	Plant-Based Drinks				
	LFM	M3.5	M1.5	F	S	H	R	C	A
Energy (kcal)	44	62	44	151	39	29	47	20	24
Fat	1.50	3.50	1.50	5.00	1.80	1.60	1.00	0.80	1.10
- Saturated fatty acid	1.00	2.30	1.00	0.60	0.30	0.20	0.10	0.80	0.10
Carbohydrates	4.70	4.70	4.70	15.60	2.50	3.20	9.50	2.70	2.70
- Sugar	4.70	4.70	4.70	9.70	2.50	3.20	3.30	1.90	2.40
Fibre	n.s.	n.s.	n.s.	0.70	0.50	0.30	0.00	0.10	0.30
Protein	3.00	3.00	3.00	9.80	3.00	0.40	0.10	0.10	0.50
Salt	0.13	0.13	0.13	0.12	0.09	0.14	0.09	0.13	0.15
Vitamin	n.s.	n.s.	n.s.	*	n.s.	n.s.	n.s.	n.s.	n.s.
- ergocalciferol (μg)	n.s.	n.s.	n.s.	n.s.	0.75	0.75	0.75	0.75	0.75
- α-tocopherol (mg)	n.s.	n.s.	n.s.	2.3	n.s.	1.8	n.s.	n.s.	n.s.
- riboflavin (mg)	n.s.	n.s.	n.s.	0.31	0.21	0.21	n.s.	n.s.	1.8
- Cyanocobalamin (μg)	n.s.	n.s.	n.s.	0.12	0.38	0.38	0.38	0.38	0.38
Minerals	n.s.	n.s.	n.s.	**	n.s.	n.s.	n.s.	n.s.	n.s.
- Calcium (mg)	120	120	120	280	120	120	120	120	120

n.s. = not stated; *: C, A, B6, B1, B3, pantothenic acid, folic acid, Biotin; ** Na, K, Cl, P, PO_4_, Mg, Fe, Zn, Cu, Mn, F, Mo, Se, Cr, I.

**Table 2:** Summary of the measured mass, water content, and disintegration time. LFM (lactose-free milk 1.5%), M3.5 (Mizo milk 3.5%), M1.5 (Mizo milk 1.5%), F (formula: Nutricia Nutridrink Diacare Vanilla flavour), S (soya), H (hazelnut), R (rice), C (coconut), and A (almond).

Code Name	Mass (mg) (*n* = 20; avg ± SD)	Water Content (%) (*n* = 5; avg ± SD)	Disintegration Time (s) (*n* = 6; avg ± SD)
LFM	161 ± 4.2	3.23 ± 0.514	35.5 ± 16.57
M3.5	189 ± 9.8	3.53 ± 0.413	74.7 ± 24.22
M1.5	155 ± 4.6	4.34 ± 0.336	21.3 ± 6.53
F	473 ± 12.2	4.17 ± 0.306	300 ± 0.00
S	135 ± 4.0	4.15 ± 0.496	12.7 ± 5.89
H	87 ± 4.7	3.59 ± 0.454	8.7 ± 3.50
C	66 ± 1.0	3.19 ± 0.599	14.0 ± 9.63
R	160 ± 3.3	3.01 ± 0.455	3.3 ± 1.63
A	76 ± 2.5	3.52 ± 0.105	26.5 ± 9.95

**Table 3:** The HPLC measurement results. LFM (lactose-free milk 1.5%), M3.5 (Mizo milk 3.5%), M1.5 (Mizo milk 1.5%), F (formula: Nutricia Nutridrink Diacare Vanilla flavour), S (soya), H (hazelnut), R (rice), C (coconut), and A (almond).

ODT Type						
		M1.5	LFM	S	H	C
Sample number	1	102.31 ± 0.421	96.98 ± 0.332	107.68 ± 0.942	105.84 ± 0.159	98.39 ± 0.438
	2	101.15 ± 0.367	94.29 ± 0.321	100.57 ± 0.137	95.64 ± 1.566	101.15 ± 0.367
	3	105.33 ± 0.733	97.55 ± 0.475	96.64 ± 0.808	97.98 ± 0.219	103.2 ± 0.178
	4	107.89 ± 1.755	93.79 ± 0.327	105.45 ± 0.626	101.81 ± 0.161	107.45 ± 0.784
	5	85.02 ± 1.143	94.35 ± 0.643	101.21 ± 0.501	100.65 ± 1.614	92.91 ± 1.363
	6	100.2 ± 0.369	90.38 ± 0.427	94.96 ± 1.132	99.82 ± 0.855	107.87 ± 0.888
	7	95.39 ± 0.467	92.08 ± 0.018	95.74 ± 0.548	105.55 ± 3.854	100.35 ± 0.237
	8	99.46 ± 0.513	101.84 ± 1.030	91.77 ± 0.589	92.92 ± 0.134	99.98 ± 0.155
	9	97.2 ± 0.279	88.29 ± 0.449	102.83 ± 0.477	97.98 ± 0.219	94.29 ± 0.944
	10	106.04 ± 1.455	90.85 ± 0.071	103.15 ± 1.508	101.81 ± 0.161	98.38 ± 0.209
	Minimum %	85.02	88.29	91.77	92.92	92.91
	Maximum %	107.89	101.84	107.68	105.84	107.87
QC	Accuracy	97.72	100.76	104.48	96.07	101.29
	Precision	2.83	0.92	1.56	3.77	1.37
	R^2^ value	0.9992	0.9945	0.9992	0.9998	0.9996

## Data Availability

The data presented in this study are openly available in the article.

## References

[1] GLOBAL REPORT—2017 Global Drug Delivery & Formulation Report: Part 1, a Global Review. https://www.proquest.com/docview/2008938105/abstract/C83043300BCB4024PQ/1.

[2] Krekeler B.N., Broadfoot C.K., Johnson S., Connor N.P., Rogus-Pulia N. (2018). Patient Adherence to Dysphagia Recommendations: A Systematic Review. Dysphagia.

[3] Blaszczyk A., Brandt N., Ashley J., Tuders N., Doles H., Stefanacci R.G. (2023). Crushed Tablet Administration for Patients with Dysphagia and Enteral Feeding: Challenges and Considerations. Drugs Aging.

[4] Marquis J., Schneider M.-P., Payot V., Cordonier A.-C., Bugnon O., Hersberger K.E., Arnet I. (2013). Swallowing Difficulties with Oral Drugs among Polypharmacy Patients Attending Community Pharmacies. Int. J. Clin. Pharm..

[5] Drumond N., Stegemann S. (2020). Better Medicines for Older Patients: Considerations between Patient Characteristics and Solid Oral Dosage Form Designs to Improve Swallowing Experience. Pharmaceutics.

[6] Király M., Sántha K., Kállai-Szabó B., Pencz K.M., Ludányi K., Kállai-Szabó N., Antal I. (2022). Development and Dissolution Study of a β-Galactosidase Containing Drinking Straw. Pharmaceutics.

[7] Kállai-Szabó N., Farkas D., Lengyel M., Basa B., Fleck C., Antal I. (2024). Microparticles and Multi-Unit Systems for Advanced Drug Delivery. Eur. J. Pharm. Sci..

[8] Cornilă A., Iurian S., Tomuță I., Porfire A. (2022). Orally Dispersible Dosage Forms for Paediatric Use: Current Knowledge and Development of Nanostructure-Based Formulations. Pharmaceutics.

[9] Kállai-Szabó N., Lengyel M., Farkas D., Barna Á.T., Fleck C., Basa B., Antal I. (2022). Review on Starter Pellets: Inert and Functional Cores. Pharmaceutics.

[10] Zajicek A., Fossler M.J., Barrett J.S., Worthington J.H., Ternik R., Charkoftaki G., Lum S., Breitkreutz J., Baltezor M., Macheras P. (2013). A Report from the Pediatric Formulations Task Force: Perspectives on the State of Child-Friendly Oral Dosage Forms. AAPS J..

[11] Montero-Padilla S., Velaga S., Morales J.O. (2017). Buccal Dosage Forms: General Considerations for Pediatric Patients. AAPS PharmSciTech.

[12] Alyami H., Koner J., Huynh C., Terry D., Mohammed A.R. (2018). Current Opinions and Recommendations of Paediatric Healthcare Professionals—The Importance of Tablets: Emerging Orally Disintegrating versus Traditional Tablets. PLoS ONE.

[13] Barbagallo M., Sacerdote P. (2018). Ibuprofen in the Treatment of Children’s Inflammatory Pain: A Clinical and Pharmacological Overview. Minerva Pediatr..

[14] Basa B., Jakab G., Kállai-Szabó N., Borbás B., Fülöp V., Balogh E., Antal I. (2021). Evaluation of Biodegradable PVA-Based 3D Printed Carriers during Dissolution. Materials.

[15] Ghourichay M.P., Kiaie S.H., Nokhodchi A., Javadzadeh Y. (2021). Formulation and Quality Control of Orally Disintegrating Tablets (ODTs): Recent Advances and Perspectives. BioMed Res. Int..

[16] Williams III R.O., Reynolds T.D., Cabelka T.D., Sykora M.A., Mahaguna V. (2002). Investigation of Excipient Type and Level on Drug Release from Controlled Release Tablets Containing HPMC. Pharm. Dev. Technol..

[17] Abrantes C.G., Duarte D., Reis C.P. (2016). An Overview of Pharmaceutical Excipients: Safe or Not Safe?. J. Pharm. Sci..

[18] Eccles R. (2020). What Is the Role of Over 100 Excipients in Over the Counter (OTC) Cough Medicines?. Lung.

[19] Dave V.S., Saoji S.D., Raut N.A., Haware R.V. (2015). Excipient Variability and Its Impact on Dosage Form Functionality. J. Pharm. Sci..

[20] Pockle R.D., Masareddy R.S., Patil A.S., Patil P.D. (2023). A Comprehensive Review on Pharmaceutical Excipients. Ther. Deliv..

[21] Elder D.P., Kuentz M., Holm R. (2016). Pharmaceutical Excipients—Quality, Regulatory and Biopharmaceutical Considerations. Eur. J. Pharm. Sci..

[22] Salim M., Eason T., Boyd B.J. (2022). Opportunities for Milk and Milk-Related Systems as ‘New’ Low-Cost Excipient Drug Delivery Materials. Adv. Drug Deliv. Rev..

[23] Gallo A., Pellegrino S., Lipari A., Pero E., Ibba F., Cacciatore S., Marzetti E., Landi F., Montalto M. (2023). Lactose Malabsorption and Intolerance: What Is the Correct Management in Older Adults?. Clin. Nutr..

[24] Misselwitz B., Pohl D., Frühauf H., Fried M., Vavricka S.R., Fox M. (2013). Lactose Malabsorption and Intolerance: Pathogenesis, Diagnosis and Treatment. United Eur. Gastroenterol. J..

[25] Dominici S., Marescotti F., Sanmartin C., Macaluso M., Taglieri I., Venturi F., Zinnai A., Facioni M.S. (2022). Lactose: Characteristics, Food and Drug-Related Applications, and Its Possible Substitutions in Meeting the Needs of People with Lactose Intolerance. Foods.

[26] Király M., Kiss B.D., Horváth P., Drahos L., Mirzahosseini A., Pálfy G., Antal I., Ludányi K. (2021). Investigating Thermal Stability Based on the Structural Changes of Lactase Enzyme by Several Orthogonal Methods. Biotechnol. Rep..

[27] Gallo A., Marzetti E., Pellegrino S., Montalto M. (2024). Lactose Malabsorption and Intolerance in Older Adults. Curr. Opin. Clin. Nutr. Metab. Care.

[28] Reyes-Jurado F., Soto-Reyes N., Dávila-Rodríguez M., Lorenzo-Leal A.C., Jiménez-Munguía M.T., Mani-López E., López-Malo A. (2023). Plant-Based Milk Alternatives: Types, Processes, Benefits, and Characteristics. Food Rev. Int..

[29] Moss R., Barker S., Falkeisen A., Gorman M., Knowles S., McSweeney M.B. (2022). An Investigation into Consumer Perception and Attitudes towards Plant-Based Alternatives to Milk. Food Res. Int..

[30] Jaeger S.R., Dupas De Matos A., Frempomaa Oduro A., Hort J. (2024). Sensory Characteristics of Plant-Based Milk Alternatives: Product Characterisation by Consumers and Drivers of Liking. Food Res. Int..

[31] Xie A., Dong Y., Liu Z., Li Z., Shao J., Li M., Yue X. (2023). A Review of Plant-Based Drinks Addressing Nutrients, Flavor, and Processing Technologies. Foods.

[32] Vilimi Z., Pápay Z.E., Basa B., Orekhova X., Kállai-Szabó N., Antal I. (2024). Microfluidic Rheology: An Innovative Method for Viscosity Measurement of Gels and Various Pharmaceuticals. Gels.

[33] Aouadi B., Zaukuu J.-L.Z., Vitális F., Bodor Z., Fehér O., Gillay Z., Bazar G., Kovacs Z. (2020). Historical Evolution and Food Control Achievements of Near Infrared Spectroscopy, Electronic Nose, and Electronic Tongue—Critical Overview. Sensors.

[34] Documentation of Ibuprofen Sodium Salt. https://www.sigmaaldrich.com/HU/en/product/sial/i1892.

[35] Siow C.R.S., Wan Sia Heng P., Chan L.W. (2016). Application of Freeze-Drying in the Development of Oral Drug Delivery Systems. Expert. Opin. Drug Deliv..

[36] Adams G., Day J.G., Stacey G.N. (2007). The Principles of Freeze-Drying. Cryopreservation and Freeze-Drying Protocols.

[37] Council of Europe 2 (2024). 9.5. Uniformity of Mass of Single-Dose Preparations. Eur. Pharmacopoeia.

[38] Council of Europe 2 (2024). 9.1. Disintegration of Tablets and Capsules. Eur. Pharmacopoeia.

[39] Council of Europe 2 (2024). 5.32. Water: Micro Determination. Eur. Pharmacopoeia.

[40] Hooper P., Lasher J., Alexander K.S., Baki G. (2016). A New Modified Wetting Test and an Alternative Disintegration Test for Orally Disintegrating Tablets. J. Pharm. Biomed. Anal..

[41] Sutthapitaksakul L., Thanawuth K., Huanbutta K., Sriamornsak P. (2022). Effect of a Superdisintegrant on Disintegration of Orally Disintegrating Tablets Determined by Simulated Wetting Test and in Vitro Disintegration Test. Pharmazie.

[42] Pabari R.M., Ramtoola Z. (2012). Effect of a Disintegration Mechanism on Wetting, Water Absorption, and Disintegration Time of Orodispersible Tablets. J. Young Pharm..

[43] Farkas D., Madarász L., Nagy Z.K., Antal I., Kállai-Szabó N. (2021). Image Analysis: A Versatile Tool in the Manufacturing and Quality Control of Pharmaceutical Dosage Forms. Pharmaceutics.

[44] Vilimi Z., Király M., Barna Á.T., Pápay Z.E., Budai L., Ludányi K., Kállai-Szabó N., Antal I. (2024). Formulation of Emulgels Containing Clotrimazole for the Treatment of Vaginal Candidiasis. Gels.

[45] Council of Europe 2 (2024). 9.6. Uniformity of Content of Single-Dose Preparations. Eur. Pharmacopoeia.

[46] Fructuoso I., Romão B., Han H., Raposo A., Ariza-Montes A., Araya-Castillo L., Zandonadi R.P. (2021). An Overview on Nutritional Aspects of Plant-Based Beverages Used as Substitutes for Cow’s Milk. Nutrients.

[47] Jeske S., Zannini E., Arendt E.K. (2017). Evaluation of Physicochemical and Glycaemic Properties of Commercial Plant-Based Milk Substitutes. Plant Foods Hum. Nutr..

[48] Craig W.J., Fresán U. (2021). International Analysis of the Nutritional Content and a Review of Health Benefits of Non-Dairy Plant-Based Beverages. Nutrients.

[49] Katidi A., Xypolitaki K., Vlassopoulos A., Kapsokefalou M. (2023). Nutritional Quality of Plant-Based Meat and Dairy Imitation Products and Comparison with Animal-Based Counterparts. Nutrients.

[50] Präger L., Simon J.C., Treudler R. (2023). Food Allergy—New Risks through Vegan Diet? Overview of New Allergen Sources and Current Data on the Potential Risk of Anaphylaxis. JDDG J. Der Dtsch. Dermatol. Ges..

[51] Daszkiewicz T., Florek M., Murawska D., Jabłońska A. (2024). A Comparison of the Quality of UHT Milk and Its Plant-Based Analogs. J. Dairy Sci..

[52] Bodor Z., Benedek C., Behling H., Kovacs Z. (2023). Fusion of Electronic Tongue and NIRS for the Detection of Heat Treatment of Honey. LWT.

[53] Podrażka M., Bączyńska E., Kundys M., Jeleń P.S., Witkowska Nery E. (2017). Electronic Tongue—A Tool for All Tastes?. Biosensors.

[54] Kazsoki A., Palcsó B., Omer S.M., Kovacs Z., Zelkó R. (2022). Formulation of Levocetirizine-Loaded Core–Shell Type Nanofibrous Orally Dissolving Webs as a Potential Alternative for Immediate Release Dosage Forms. Pharmaceutics.

[55] Kovacs Z., Szöllősi D., Zaukuu J.-L.Z., Bodor Z., Vitális F., Aouadi B., Zsom-Muha V., Gillay Z. (2020). Factors Influencing the Long-Term Stability of Electronic Tongue and Application of Improved Drift Correction Methods. Biosensors.

[56] Németh D., Balázs G., Daood H.G., Kovács Z., Bodor Z., Zinia Zaukuu J.-L., Szentpéteri V., Kókai Z., Kappel N. (2019). Standard Analytical Methods, Sensory Evaluation, NIRS and Electronic Tongue for Sensing Taste Attributes of Different Melon Varieties. Sensors.

[57] Imam M., Nagpal K. (2023). The Electronic Tongue: An Advanced Taste-Sensing Multichannel Sensory Tool with Global Selectivity for Application in the Pharmaceutical and Food Industry. Pharm. Dev. Technol..

